# Hydroxychloride trace minerals have a positive effect on growth performance, carcass quality and impact ileal and cecal microbiota in broiler chickens

**DOI:** 10.1186/s40104-021-00553-7

**Published:** 2021-03-09

**Authors:** Sandra J. A. van Kuijk, Yanming Han, Ana Isabel Garcia-Ruiz, Ana Rodiles

**Affiliations:** 1 Trouw Nutrition R&D, P.O. Box 299, 3800 AG Amersfoort, the Netherlands; 2 Trouw Nutrition R&D, 45950 Casarrubios del Monte, Toledo Spain

**Keywords:** Broiler chickens, Growth performance, Gut microbiota, Hydroxychloride trace minerals

## Abstract

**Background:**

The objective was to study the effect of hydroxychloride trace minerals (HTM) on growth performance, carcass quality and gut microbiota of broiler chickens in comparison to sulphate trace minerals (STM). In total 1440 male Ross 308 day-old chicks were divided into 12 replicate pens with 30 birds each per treatment. Four different treatments were tested according to a 2 × 2 factorial study design, where the animals received a three phase diet containing either inorganic Zn from sulphates or Zn from HTM in high (80 mg/kg Zn) or low Zn dosage (20 mg/kg Zn). In all treatments 15 mg/kg Cu was added from the same mineral source as the Zn. Body weight and feed intake were measured on day 0, 10, 27 and 34, while carcass and breast meat yields were measured at the end of the study (day 34). In addition, high-throughput sequencing analysis was performed in digesta samples from ileum and cecum to study the gut microbiome (day 34).

**Results:**

The results showed an improved (*P* < 0.05) body weight of broiler chickens fed HTM, regardless of Zn level, on day 27, while on day 34 this effect remained as a tendency (*P* = 0.0542). In the overall study period, birds fed HTM had a higher (*P* < 0.05) average daily gain and average daily feed intake when compared to birds fed STM. The mineral source did not affect the carcass characteristics, however, feeding 80 mg/kg Zn resulted in a significantly higher (*P* = 0.0171) breast meat yield, regardless of source. High-throughput sequencing analysis of the microbiota revealed a higher microbial diversity in the ileum and cecum of HTM fed birds compared to STM fed birds. Taxonomical differences were mainly found in the cecum, specifically between the group fed high and low Zn levels from HTM. This correlated with the mineral contents observed in the cecal digesta. Comparing both groups fed 80 mg/kg Zn, the HTM group had more Streptococcaceae, *Streptococcus*, Clostridia, *Weissella* and Leuconostocaceae compared to the STM group.

**Conclusions:**

HTM improved growth performance of broiler chickens; and the source and level of Zn modulated the gut microbiota communities in broilers differentially.

## Background

Zinc is an essential trace mineral for broiler chickens. Zinc has been proven to play a role in immunity, enzymatic processes and eventually in growth [1, 2]. Therefore, Zn deficiency can cause major problems. To prevent a deficiency, it is a commercial practice to add Zn to diets at higher dosages than those advised for poultry by the National Research Council (NRC) [3]. Higher dosages in the diet will however result in more excretion of this trace mineral into the environment. Recently, the EFSA (European food safety agency) published guidelines about the importance of reducing the amount of heavy metals into the environment [4]. Thus, it is essential to reduce the amount of Zn added to broiler diets. As expected, reducing the dose in the diet will reduce the excretion into the environment [5]. Inorganic trace minerals in the form of sulphates have a lower bioavailability than other commercially available mineral sources [6]. Hydroxychloride trace minerals (HTM) are a good example of a more bioavailable mineral source in broilers compared to sulphate trace minerals (STM) [7]. In addition, HTM do have a unique crystalline structure that is not readily soluble at a pH above 4 [8, 9]. Moreover, HTM are much less reactive in the feed than STM, and do not interact with antinutritional factors such as phytate [10, 11]. In addition, the low solubility at higher pH suggests that the pH in the gastrointestinal tract may have a specific effect on the function of HTM. Both differences in solubility and reactivity between HTM and STM may result in different quantities and forms of Zn present in the different compartments of the gastrointestinal tract. It is also known that Zn is essential for several microbes [12], indicating that a different source and level of Zn available in the gut may influence the microbiota. Olukosi et al. [13] already showed a beneficial effect of HTM on growth performance and carcass quality in broiler chickens. The objective of the present study was to investigate the effects on growth performance, carcass and breast meat yield, and microbiota of broiler chickens fed STM or HTM in high and low Zn dosages in presence of 15 mg/kg Cu of the same mineral source. With the above mentioned differences in solubility and bioavailability, we expected a larger contrast between high and low Zn levels in HTM fed birds compared to STM fed birds.

## Materials and methods

### Animals and dietary treatments

In total, 1440 newly hatched male Ross 308 broiler chicks were housed at the Poultry Research Facility of Trouw Nutrition, Casarrubios del Monte, Spain. The birds were randomly allocated into groups of 30 animals per floor pen (3 m × 1 m) with 6 and 18 h of light/dark periods. Each pen provided 0.10 m^2^/bird with wood shavings litter. Each group was randomly allocated to one of four treatments with 12 replicate pens each. The four treatments were applied according to a 2 × 2 factorial study design, in which two mineral sources of zinc (STM and HTM) were tested at two different zinc levels (high: 80 mg/kg and low: 20 mg/kg), whereas to all diets 15 mg/kg Cu was added from the same mineral source as the Zn. Sulphate trace minerals source were added to the feed in the form of Zn sulphate (zinc sulphate monohydrate, Sigma-Aldrich, Saint Louis, MO, USA), and Cu sulphate (copper(II) sulphate pentahydrate, Sigma-Aldrich, Saint Louis, MO, USA). Hydroxychloride trace minerals were added at the same Zn (IntelliBond Z, Trouw Nutrition, The Netherlands) and Cu (IntelliBond C, Trouw Nutrition, The Netherlands) levels as for the sulphate minerals. The composition of the diets is shown in Table 1. The wheat-soybean meal diets were offered as pellets in three phases, with phase 1 being from day 0 to 10, phase 2 from day 10 to 27 and phase 3 from day 27 to 34, according to the NRC requirements of the species [3]. The analysed mineral contents of the diets are shown in Table 2. Animals were fed ad libitum throughout the entire study period of 34 days.

**Table 1:** Feed formulation used in the three feeding phases

Ingredient, %	Starter	Grower	Finisher
Wheat	53.511	65.490	67.949
Soya bean meal	29.149	25.267	22.554
Soya oil	6.969	5.177	5.819
Wheat bran	5.000		
Potato protein	1.459	1.334	1.400
Calcium-carbonate fine	1.206	0.566	0.455
Monocalcium phosphate	1.072	0.553	0.261
Vit-min premix without Zn and Cu^1^	0.500	0.500	0.500
Sodium bicarbonate	0.256	0.221	0.210
*DL*-Methionine	0.249	0.230	0.213
*L*-Lysine HCl	0.224	0.240	0.215
Salt (NaCl)	0.166	0.166	0.174
Xylanase	0.100	0.100	0.100
Phytase	0.100	0.100	0.100
*L*-Threonine	0.038	0.056	0.051
Calculated AME broilers, kcal	2850	2925	3000
Analysed nutrients			
Crude protein, g/100 g	21.5	19.6	18.9
Crude ash, g/100 g	5.0	3.8	3.5
Crude fiber, g/100 g	2.8	2.7	2.6
Moisture, g/100 g	8.4	8.8	8.7
Ca, g/100 g	0.90	0.58	0.48
P, g/100 g	0.61	0.46	0.38

^1^Provided per kg of final feed: 0.65 g calcium, 90 mg manganese oxide, 1 mg potassium iodide, 0.25 mg selenium (sodium selenite), 65 mg iron (ferrous carbonate), 10,000 IU vitamin A, 2500 IU vitamin D_3_, 50 UI vitamin E (all-rac-α-tocopherol-acetate), 2 mg vitamin K_3_, 2 mg vitamin B_1_, 6 mg vitamin B_2_, 4 mg vitamin B_6_ (pyridoxine hydroxychloride), 25 μg vitamin B_12_, 40 mg niacinamide, 10 mg calcium *D*-pantothenate, 10 mg pantothenic acid, 1 mg folic acid, 150 μg biotin, 300 mg choline chloride, 0.61 g sepiolite, 0.75 mg butylated hydroxyanisole (BHA), 8.25 mg butylated hydroxytoluene (BHT), 1.20 mg ethoxyquin

**Table 2:** Analysed copper and zinc levels in the experimental diets (hydroxychloride trace minerals, HTM and sulphate trace minerals, STM) in high and low dosage

Diet	Copper, mg/kg	Zinc, mg/kg
Starter phase 1		
STM high	23	116
STM low	20	60
HTM high	24	120
HTM low	22	60
Grower phase 2		
STM high	22	114
STM low	22	56
HTM high	22	118
HTM low	21	54
Finisher phase 3		
STM high	21	112
STM low	22	52
HTM high	22	117
HTM low	22	52

At the end of the experiment, 6 animals per pen were selected based on average body weight and euthanized by cervical dislocation according to the guidelines of the Ethics Committee of Poultry Research Centre of Trouw Nutrition for the humane care and use of animals in research. Animal procedures were verified by the External Ethical Committee of “Hospital General Universitario de Ciudad Real”, and approved by the Junta de Castilla-La Mancha Animal Welfare department as compliable with the RD 53/2013 of the 1st of February which establishes basic rules applied to protect animals used for research, and other scientific matters like teaching. The present study was approved by the Trouw Nutrition Animal Care Committee and followed recommendations of the Castilla-La Mancha Animal Welfare Department (Royal degree RD 53/2013), in compliance with the European Union guidelines for the care and use of animals in research (European Parliament, 2010). From the six birds selected per pen, five birds were used for carcass and breast meat yields and the remaining one was sampled for both the microbiota and mineral content. Samples were collected aseptically by opening the abdominal cavity with a sterile blade, the intestinal content (digesta) from the ileum and cecum was placed into a sterile tube until further analysis. All samples were immediately snap frozen on dry ice and then transferred to a − 80 °C freezer.

### Zootechnical parameters

Body weight and feed intake was measured per pen at the start of the experiment at day 0 and at the end of each feeding phase at day 10, 27 and 34. The body weight and feed intake measures were used to calculate the average daily gain, the average daily feed intake and the feed conversion ratio (feed:gain). On day 34, the carcasses were weighted and both breast muscles were excised and weighted by qualified personnel. These weights were used to calculate carcass and breast yield, as the percentage of the live weight or the carcass weight, respectively.

### Mineral content of ileum and cecum digesta

Zinc and copper content in the diets and in 1 ileal and 1 cecal digesta samples (from the same animal) per pen, resulting in 12 ileal and 12 cecal samples per treatment were analysed according to ISO (ANA-00281) using inductively coupled plasma-mass spectroscopy (ICP-MS) (Nexion 300D, Perkin elmer, Waltham, MA, USA) with an autosampler coupled (4DX, Elemental Scientific (ESI), Omaha, NE, USA). The chemicals used were nitric acid (67–69%) and hydrochloric acid (34–35%) of ultra pure grade for trace mineral analysis (VWR, Amsterdam, The Netherlands).

### DNA extraction, PCR and library preparation

From the ileal and cecal digesta 200 mg of samples were lysated with MagNA Lyser (Roche, Burges Hill, UK) prior to DNA extraction. DNA extraction was performed with PowerMicrobiome™ RNA isolation kit (MO BIO, Carlsbad, CA, USA) following the manufacturer’s instructions with some modifications, such as omitting the β-mercaptoethanol and DNase I steps. The concentration of the extracted prokaryotic DNA in each sample was calculated by qPCR with 926F [14] and 1027R [15] primers at a concentration of 0.4 μmol/L in iQ SYBRgreen Supermix qPCR (Bio-Rad Laboratories Inc., Hercules, CA, USA). PCR was carried out with Universal primers 341F-785R of V3-V4 regions to amplify 16S rRNA in a dual-index sequencing strategy according to [16] with Taq KAPA HiFi Hotstart ReadyMix (Kapa Biosystems, Woburn, MA, USA) and 12.5 ng bacterial DNA to reduce PCR bias. Four PCR products from the same sample were pooled before cleaning up step with QIAquick Gel Extraction Kit (Qiagen, Hilden, Germany). Negative controls and MOCK communities were included in the sequencing as controls. The library was sequenced on an Illumina HiSeq platform 2 × 300 paired end. All reagents used were molecular grade.

### High-throughput sequencing and bioinformatics analysis

Raw fastq files were imported, demultiplexed and processed using QIIME 2 (version 2018.6) [17]. Paired-ends fastq files were quality filtered and dereplicated with DADA2 [18]. Alpha and beta diversity were calculated under rooted phylogeny downsampling to the lowest count of sequences. Taxonomy was assigned to the resulting 16S rRNA marker genes against the SILVA (version 132) 99% OTUs (trained with naive-bayes for 341F-785R region of the 16S) using sklearn classifier method to determine the taxonomy according to Bokulich et al. [19]. Chloroplast and mitochondria were considered as contamination and removed from the otus_biom table, as well as low frequency OTUs (< 10 reads in < 5 samples), previously to statistical analysis.

### Statistical analysis

The pen was the experimental unit for all parameters. Growth performance data were analysed according to the 2 × 2 factorial design using the MIXED procedure in SAS software (version 2012, SAS Institute Inc. Cary, NC, USA). The model included source and level and feeding phase as fixed effects. The different time points were included as repeated measures. The carcass and breast meat yield were subjected to the GLIMMIX procedure in SAS. Post hoc analysis was done using Tukey. R in QIIME2 (version 2018.6) was used to analyse alpha diversity with Kruskal-Wallis and Spearman for correlations, beta diversity with PERMANOVA and ANOSIM with a sequencing depth of 234,000 and 175,000 sequences for ileum and cecum, respectively. Taxonomy data was analysed with LEfSE [20] and represented in RDA plots with Canoco. Microbiota results were analysed comparing the four treatments, and in addition the main effects of mineral source and level were explored. The results were considered being significantly different when *P* < 0.05.

## Results

### Growth performance and carcass characteristics

Growth performance results are shown in Table 3. At day 27 of the study, birds fed HTM had a significantly (*P* = 0.0229) higher body weight than birds fed STM, regardless of mineral levels. This continued until day 34 as a strong trend (*P* = 0.0542) towards an increased body weight in birds fed HTM compared to birds fed STM. Average daily gain (ADG) and average daily feed intake (ADFI) followed the same patterns as body weight throughout the study. In this case, between 10 and 27 days a tendency was observed towards an increased ADG (*P* = 0.0738) and ADFI (*P* = 0.0846) for birds fed HTM compared to birds fed STM. However, this tendency resulted in a significantly improved ADG (*P* = 0.0130) and ADFI (*P* = 0.0125) over the entire period for HTM fed birds compared to STM fed birds, regardless of mineral level. As a result, the feed conversion ratio (FCR) was not significantly different among treatments.

**Table 3:** Body weight (BW), average daily gain (ADG), average daily feed intake (ADFI) and feed conversion ratio (FCR) of the birds fed with the experimental diets (hydroxychloride trace minerals, HTM and sulphate trace minerals, STM) in high and low dosages (± standard error)

		STM		HTM		*P*-value		
	Day	High	Low	High	Low	Source	Level	Source× Level
BW, g	0	43.2 ± 0.2	43.2 ± 0.1	43.5 ± 0.3	43.2 ± 0.2	0.3240	0.3747	0.4937
	10	275.7 ± 4.1	278.2 ± 2.0	277.6 ± 3.0	279.1 ± 2.0	0.5471	0.3927	0.7652
	27	1774.0 ± 12.7	1787.4 ± 12.2	1810.2 ± 168	1806.1 ± 9.9	**0.0229**	0.6872	0.1112
	34	2463.7 ± 13.5	2476.0 ± 15.3	2506.9 ± 21.6	2493.1 ± 18.3	**0.0542**	0.9657	0.2137
ADG, g/(bird·d)	0–10	23.3 ± 0.4	23.5 ± 0.2	23.4 ± 0.3	23.6 ± 0.2	0.6950	0.4004	0.8352
	10–27	88.2 ± 0.6	88.8 ± 0.6	90.2 ± 0.8	89.8 ± 0.5	**0.0738**	0.8970	0.3155
	27–34	98.7 ± 0.8	98.3 ± 0.9	99.5 ± 1.2	98.2 ± 1.4	0.7133	0.4015	0.7773
	0–34	71.0 ± 0.4	71.6 ± 0.5	72.5 ± 0.6	72.2 ± 0.5	**0.0130**	0.7071	0.3407
ADFI, g/(bird·d)	0–10	25.1 ± 0.4	25.2 ± 0.3	25.1 ± 0.4	25.4 ± 0.3	0.6246	0.4419	0.7749
	10–27	121.2 ± 0.8	122.0 ± 0.9	123.4 ± 1.2	123.8 ± 1.0	**0.0846**	0.6205	0.3499
	27–34	156.8 ± 1.8	157.0 ± 1.1	158.3 ± 1.0	156.0 ± 1.7	0.8866	0.4835	0.7457
	0–34	99.8 ± 0.6	100.6 ± 0.6	101.5 ± 0.8	101.5 ± 0.7	**0.0125**	0.4312	0.4505
FCR	0–10	1.088 ± 0.007	1.071 ± 0.001	1.073 ± 0.015	1.078 ± 0.012	0.7182	0.6065	0.7631
	10–27	1.374 ± 0.006	1.374 ± 0.004	1.369 ± 0.004	1.378 ± 0.006	0.9621	0.6985	0.9611
	27–34	1.590 ± 0.020	1.597 ± 0.010	1.593 ± 0.016	1.591 ± 0.026	0.8683	0.8420	0.9797
	0–34	1.406 ± 0.008	1.406 ± 0.034	1.402 ± 0.005	1.407 ± 0.008	0.7415	0.6981	0.6875

*P*-values below 0.1 are bolded

The effect of the treatments on carcass and breast meat yield were measured at the end of the study (Table 4). No significant differences were found in carcass yield. Breast meat yield was significantly higher in birds fed 80 mg/kg Zn compared to birds fed 20 mg/kg Zn (*P* = 0.0171), while no significant differences were found between the sources or any source×level interaction.

**Table 4:** Carcass yield and breast meat yield of the birds fed with the experimental diets (hydroxychloride trace minerals, HTM and sulphate trace minerals, STM) in high and low dosages

	STM		HTM		*P*-value		
	High	Low	High	Low	Source	Level	Source×Level
Carcass yield, % of body weight	66.8 ± 0.19	66.5 ± 0.22	67.2 ± 0.22	66.9 ± 0.29	0.1239	0.1956	0.8997
Breast meat yield, % of carcass weight	27.8 ± 0.21	27.3 ± 0.23	28.1 ± 0.23	27.4 ± 0.26	0.3906	**0.0171**	0.5172

*P*-values below 0.1 are bolded

### Mineral content in ileal and cecal digesta

The mineral content in the digesta from ileum and cecum is shown in Table 5. Birds fed HTM had significantly higher Cu contents in the ileum compared to the birds fed STM (*P* = 0.004). The Zn content in the ileum was proportional to the dietary Zn level, where birds fed a high dietary Zn presented significantly higher Zn than in birds fed low Zn (*P* < 0.0001). In the cecum, the Cu level remained similar across the treatments, while Zn content depended significantly on the supplemented levels (*P* < 0.0001). Surprisingly, there was a significant source×level interaction where birds fed 80 and 20 mg/kg Zn from HTM showed the highest and the lowest cecum values, respectively (*P* = 0.0472).

**Table 5:** Mineral contents in ileal and cecal digesta at day 34 of the birds fed with the experimental diets (hydroxychloride trace minerals, HTM and sulphate trace minerals, STM) in high and low dosages

	STM		HTM		*P*-value		
	High	Low	High	Low	Source	Level	Source×Level
Ileum							
Cu	11.0 ± 0.97	10.4 ± 0.66	13.0 ± 0.99	12.8 ± 0.52	**0.0040**	0.5539	0.7942
Zn	52.3 ± 5.60	24.2 ± 2.23	59.2 ± 6.67	26.8 ± 1.48	0.2020	**< 0.0001**	0.5616
Cecum							
Cu	89.9 ± 11.42	96.1 ± 10.34	89.2 ± 5.45	92.0 ± 15.59	0.8163	0.6569	0.8671
Zn	175.7 ± 24.66^ab^	117.7 ± 14.16^bc^	224.8 ± 21.97^a^	102.4 ± 21.71^c^	0.2896	**< 0.0001**	**0.0472**

^a,b,c^ different superscripts inside of each row represent significant differences among treatments based on the source×level interaction effect (*P* < 0.05). *P*-values below 0.1 are bolded

### Microbiota

**Fig. 1:**
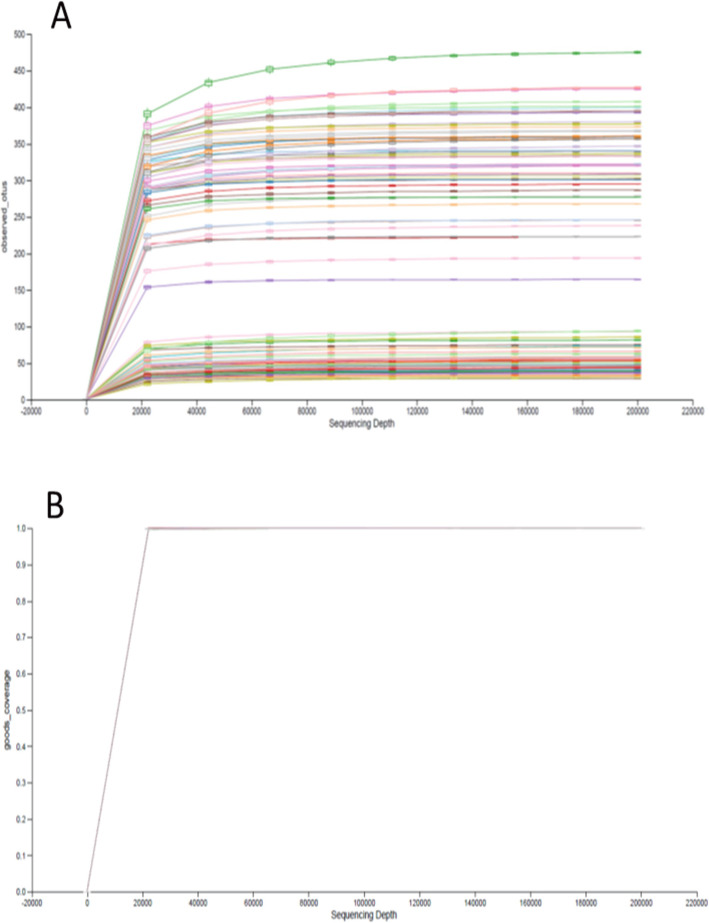
Alpha rarefaction of observed OTUs (**a**) and good coverages (**b**) in all the samples at day 34

In total, 42,854,714 sequences were obtained after removal of chloroplast and mitochondria reads (6046 sequences). Rarefactions curves of observed OTUs showed a *plateau* (Fig. 1) and good coverage values close to 1 from 20,000 sequences (0.9999 ± 0.0001), showing that sampling depth for all the members of the microbial communities were sampled. Alpha and beta diversity showed a clear (and significant) separation between the ileum and cecum (data not shown).

In the ileum, 23,998,507 sequences were recovered (521,707 ± 174,002 sequences) representing 100 OTUs. Beta diversity ordination represented by redundancy analysis (Fig. 1) showed significant distances on ANOSIM (*P* = 0.020) and PERMANOVA (*P* = 0.014) of unweighted unifrac and a trend in jaccard (*P* = 0.080), between microbial communities in the ileum of birds fed 80 mg/kg Zn from HTM to birds fed 20 mg/kg from HTM. The STM fed birds showed intermediate values not differing from any of the other treatments tested. The number of observed OTUs of alpha diversity was significantly higher in the group fed 80 mg/kg Zn from HTM (50 ± 14.1) than 20 mg/kg Zn from HTM (39 ± 8.3) (*P* = 0.049), as well as the phylogenetic distances, whereas both STM groups were in-between (Table 6). In addition, Spearman correlations showed a tendency towards a positive correlation between the number of observed species in the ileum and the breast meat yield (*P* = 0.093) (data not shown). The taxonomy at phyla level (data not shown) showed Firmicutes as main phyla (99.8%), followed by Actinobacteria (0.15%), Proteobacteria (0.08%) and Patescibacteria (0.01%). Actinobacteria phyla was significantly higher in group fed 80 mg/kg Zn from HTM (0.34%) in comparison with the group fed 20 mg/kg Zn from HTM (0.03%) (*P* = 0.043). At genus level, the main organism present in the ileum was *Lactobacillus* (> 88%) (Table 7).

**Table 6:** Alpha diversity parameters in the ileum and cecum (*n* = 12) at day 34 of the birds fed with the experimental diets (hydroxychloride trace minerals, HTM and sulphate trace minerals, STM) in high and low dosages

Gut section	Source	Level	Observed_OTUS	Shannon	Evenness	Faith_pd
Ileum	STM	High	43 ± 10^ab^	2.6 ± 0.6	0.48 ± 0.09	4.0 ± 0.8^ab^
		Low	44 ± 9^ab^	2.3 ± 0.6	0.43 ± 0.13	4.1 ± 1.2^ab^
	HTM	High	50 ± 14^a^	2.8 ± 0.6	0.50 ± 0.11	4.4 ± 0.9^a^
		Low	39 ± 8^b^	2.5 ± 0.5	0.48 ± 0.09	3.6 ± 0.7^b^
*P*-value main effects		Source	0.8602	0.3557	0.4680	0.7085
		Level	0.1133	0.1440	0.4100	0.1112
Cecum	STM	High	288 ± 62	5.6 ± 0.4^b^	0.69 ± 0.03	18 ± 2^b^
		Low	308 ± 67	5.8 ± 0.4^ab^	0.70 ± 0.03	19 ± 2^ab^
	HTM	High	312 ± 41	5.9 ± 0.3^ab^	0.71 ± 0.03	19 ± 1^ab^
		Low	327 ± 29	6.0 ± 0.2^a^	0.71 ± 0.02	20 ± 1^a^
*P*-value main effects		Source	0.3588	0.0665	0.0990	0.3025
		Level	0.1270	0.3325	0.7887	**0.0696**

^a,b^ Different superscripts inside of each column represents significant differences among treatments per region based on pairwise comparisons (*P* < 0.05). *P*-values below 0.1 are bolded

**Table 7:** Genus composition of the ileum at day 35 (*n* = 12) at day 34 of the birds fed with the experimental diets (hydroxychloride trace minerals, HTM and sulphate trace minerals, STM) in high (80 mg/kg) and low (20 mg/kg) dosages

	STM		HTM	
	High	Low	High	Low
*Lactobacillus*	92.2 ± 9.5^ab^	98.0 ± 3.8^a^	88.8 ± 11.08^b^	96.2 ± 7.97^ab^
*Enterococcus*	6.10 ± 8.60^ab^	0.852 ± 2.24^b^	7.7 ± 8.2^a^	3.08 ± 7.29^ab^
*Streptococcus*	0.743 ± 0.868^ab^	0.429 ± 1.01^b^	2.4 ± 3.5^a^	0.385 ± 0.800^b^
*Candidatus Arthromitus*	0.647 ± 1.354	0.316 ± 0.534	0.113 ± 0.170	0.288 ± 0.544
*Corynebacterium 1*	0.071 ± 0.084	0.127 ± 0.133	0.250 ± 0.342	0.018 ± 0.016
*Staphylococcus*	0.050 ± 0.063	0.078 ± 0.098	0.378 ± 0.603	0.024 ± 0.019
*Escherichia-Shigella*	0.073 ± 0.142	0.033 ± 0.076	0.197 ± 0.477	0.015 ± 0.018
*Weissella*	0.006 ± 0.010^ab^	0.023 ± 0.045^ab^	0.021 ± 0.030^a^	0.001 ± 0.001^b^
*Bifidobacterium*	0.007 ± 0.008^ab^	0.014 ± 0.016^ab^	0.039 ± 0.053^a^	0.008 ± 0.015^b^
*Brachybacterium*	0.026 ± 0.065	0.025 ± 0.042	0.037 ± 0.080	0.000 ± 0.000
*Aerococcus*	0.010 ± 0.033	0.017 ± 0.034	0.011 ± 0.017	0.000 ± 0.000
*Clostridium* sensu stricto *1*	0.018 ± 0.050	0.004 ± 0.007	0.018 ± 0.057	0.004 ± 0.011
*Blautia*	0.001 ± 0.001^ab^	0.003 ± 0.006^ab^	0.007 ± 0.009^a^	0.001 ± 0.002^b^
*Dietzia*	0.000 ± 0.001	0.002 ± 0.004	0.002 ± 0.004	0.000 ± 0.000
*Brevibacterium*	0.007 ± 0.021	0.006 ± 0.015	0.007 ± 0.019	0.000 ± 0.000
*Ruminococcus torques g*	0.001 ± 0.002^ab^	0.003 ± 0.003^ab^	0.004 ± 0.005^a^	0.002 ± 0.006^b^
*Faecalibacterium*	0.001 ± 0.001	0.004 ± 0.006	0.002 ± 0.003	0.003 ± 0.009
*Pediococcus*	0.001 ± 0.001	0.000 ± 0.000	0.002 ± 0.004	0.000 ± 0.002
*Lachnospiraceae_unknown*	0.002 ± 0.004	0.001 ± 0.003	0.003 ± 0.005	0.000 ± 0.001
*Jeotgalicoccus*	0.006 ± 0.017	0.008 ± 0.013	0.009 ± 0.016	0.000 ± 0.000
*Facklamia*	0.001 ± 0.003	0.001 ± 0.003	0.002 ± 0.005	0.000 ± 0.000
*Eubacterium hallii g*	0.000 ± 0.000^ab^	0.000 ± 0.001^ab^	0.001 ± 0.001^a^	0.000 ± 0.000^b^
*Anaerostipes*	0.000 ± 0.000	0.000 ± 0.000	0.000 ± 0.000	0.000 ± 0.001
*CAG-56*	0.000 ± 0.000	0.000 ± 0.000	0.000 ± 0.001	0.000 ± 0.001
*Sellimonas*	0.000 ± 0.001	0.000 ± 0.001	0.001 ± 0.002	0.000 ± 0.002
*Saccharimonadales_unknown*	0.000 ± 0.001	0.001 ± 0.002	0.000 ± 0.000	0.000 ± 0.000
*Paracoccus*	0.000 ± 0.000	0.000 ± 0.001	0.000 ± 0.000	0.000 ± 0.000

^a,b^ Different superscripts inside of each row represents significant differences among treatments (*P* < 0.05)

**Fig. 2:**
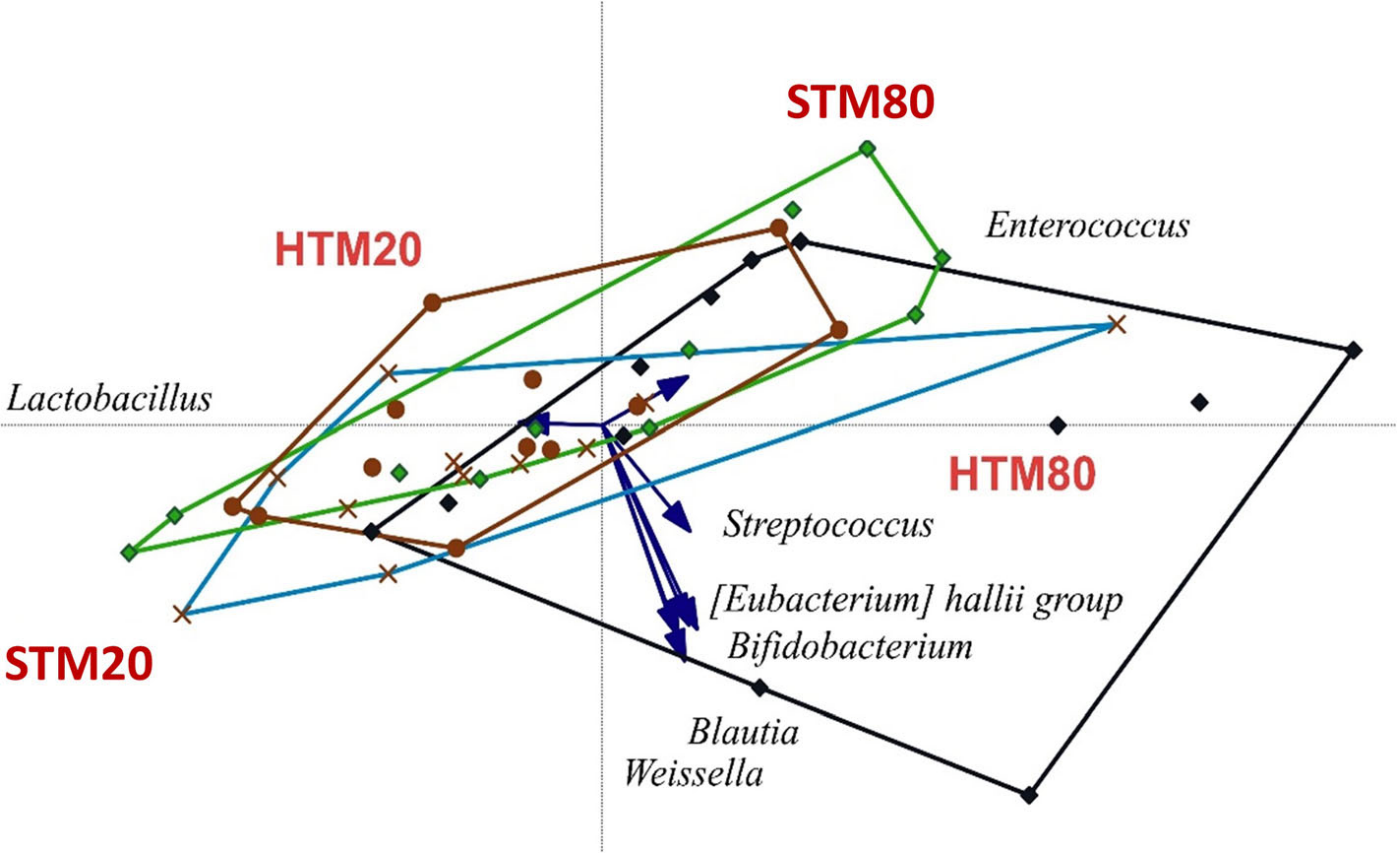
Redundancy analysis (RDA) of the ileum at day 34. Significant differences are represented by the arrows. HTM20: 20 mg/kg Zn from hydroxychloride trace minerals (HTM), HTM80: 80 mg/kg Zn from HTM, STM20: 20 mg/kg Zn from sulphate trace minerals (STM), STM80: 80 mg/kg Zn from STM

Differences in the remaining genera in the ileum are shown in Table 7 and visualized in Fig. 2. The different areas show that the diet containing 80 mg/kg Zn from HTM permitted some diversity strength in the ileum at day 34. The arrows in Fig. 2 show significantly increased *Enterococcus* (*P* = 0.012) and *Streptococcus* (*P* = 0.042), in comparison to the diet containing 20 mg/kg Zn from STM, which in turn presented higher *Lactobacillus* than 80 mg/kg Zn from HTM (*P* = 0.010). The group fed 80 mg/kg Zn from HTM also presented higher *Bifidobacterium* (*P* = 0.018), *Blautia* (*P* = 0.034), *Streptococcus* (*P* = 0.043), *Weissella* (*P* = 0.039), *Eubacterium hallii* group (*P* = 0.033), *Ruminococcus torques g* (*P* = 0.043) than the group fed 20 mg/kg Zn from HTM (Table 7). Dosage levels (20 versus 80 mg/kg) presented significant differences of abundance in *Lactobacillus* (20 mg/kg) and in *Streptococcus* and *Enterococcus* (80 mg/kg), mainly because of the high influence of the group fed 80 mg/kg Zn from HTM. No significant difference was found by the overall source effect, regardless of Zn level.

**Fig. 3:**
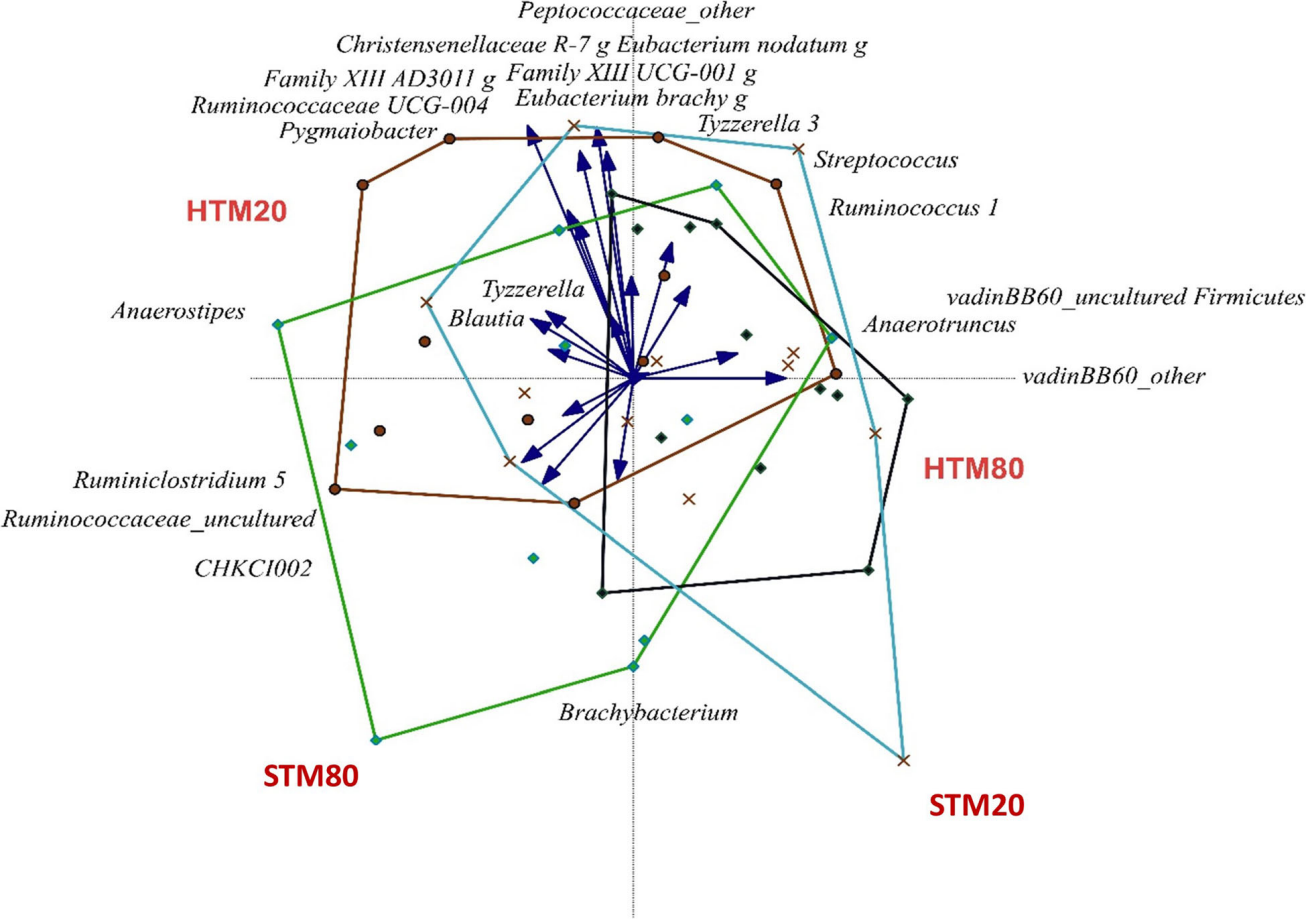
Redundancy analysis (RDA) of the cecum at day 34. Significant differences are represented by the arrows. HTM20: 20 mg/kg Zn from hydroxychloride trace minerals (HTM), HTM80: 80 mg/kg Zn from HTM, STM20: 20 mg/kg Zn from sulphate trace minerals (STM), STM80: 80 mg/kg Zn from STM

In the cecum, 17,250,171 sequences were recovered (359,379 ± 111,998 sequences) representing 644 OTUs. Beta diversity ordination represented by redundancy analysis (Fig. 3) showed significant distances in ANOSIM per source of the unweighted unifrac and jaccard distances (*P* = 0.019 and *P* = 0.027, respectively), promoted by significant differences between both groups fed 20 mg/kg Zn (*P* = 0.007 and *P* = 0.031, respectively). Feeding 20 mg/kg Zn from HTM also presented marked differences in the ANOSIM and PERMANOVA of unweighted unifrac distances in comparison with feeding 80 mg/kg Zn from HTM (*P* = 0.014 and *P* = 0.011, respectively) or from STM (*P* = 0.011 and *P* = 0.022, respectively). The alpha diversity confirmed the differences found in the ordination of the bacterial communities (see Table 6). The Shannon index, an indication for population diversity, shows a tendency towards a higher diversity in the HTM fed groups compared to the STM groups (*P* = 0.066), with the group fed 80 mg/kg Zn from STM having the lowest diversity in comparison with the group fed 20 mg/kg Zn from HTM (*P* = 0.049). The observed species in the cecum tended to be higher in birds fed 20 mg/kg Zn from HTM compared to the birds fed 80 mg/kg Zn from STM (*P* = 0.067). The evenness showed a trend towards an increased (*P* = 0.099) richness in the HTM groups compared to the STM groups, regardless of Zn level. Taking the phylogenetic distances into account, the Faith_PD parameter did show a trend towards a different community in the groups fed 20 mg/kg Zn compared to the groups fed 80 mg/kg Zn (*P* = 0.070), and specifically between 20 mg/kg Zn from HTM and 80 mg/kg Zn from STM (*P* = 0.024) (Table 6). In addition, significant positives correlations were found between the carcass yield and Shannon (*P* = 0.0322), evenness (*P* = 0.046) or observed species (trend, *P* = 0.062) in the cecum. Also the ADFI and ADG performance indexes tended to be positively correlated to the richness or evenness of the microbiota (*P* = 0.053 and *P* = 0.098, respectively) (data not shown).

The taxonomy in the cecum presented as the main phylum Firmicutes (87.3%), Actinobacteria (9.5%), Bacteroidetes (3.1%), and in less extend Proteobacteria (0.06%), Tenericutes (0.05%) and Cyanobacteria (0.01%). On family level, the main taxa present in the cecum were the Lachnospiraceae, Ruminococcaceae, Lactobacillaceae and Bifidobacteriaceae.

At genus level, the main organisms present in the cecum were *Lactobacillus*, *Faecalibacterium*, unknown genus from Lachnospiraceae family, *Bifidobacterium*, several *Ruminococcus* groups, *Butyricoccus*, *Blautia* and *Alistipes* (Table 8  and Fig. 3). Being 80 mg/kg the most common commercial level used in broiler feed the most interesting comparison would be between groups fed 80 mg/kg Zn from either STM or HTM. In this case, significant differences were found in *Streptococcus* and two unidentified genus from the family vadin BB60 being higher in birds fed 80 mg/kg Zn from HTM than from STM. Only *CHKCI002* from Eggerthellaceae family presented higher values in birds fed 80 mg/kg Zn from STM than from HTM. As in the ileum, the majority of the significant differences were in between HTM groups (Table 8). The group fed 20 mg/kg Zn from HTM contained higher relative abundances of *Blautia*, *Anaerostipes*, Christensenellaceae R-7 group, *Ruminococcus* 1, Ruminococcaceae UCG-004, *Tyzzerella*, *Eubacterium nodatum* group, Ruminococcaceae_uncultured, Family XIII (*AD3011* and *UCG-001* groups), *Eubacterium brachy* group, *Pygmaiobacter*, Peptococcaceae_other, whereas the group fed 80 mg/kg Zn from HTM showed significantly higher relative amounts of vadin BB60 group (Table 8). The group fed 20 mg/kg Zn from STM reached the highest *Anaerotruncus* level, *Tyzzerella* 3, Family XIII *UCG-001*, and *Brachybacterium*, and the group fed 80 mg/kg Zn from STM the highest *CHKCI002* (Table 8). Dosage level 20 mg/kg presented higher significant abundance of *Anaerostipes*, *Pygmaiobacter*, unknown genus from Peptococcaceae family, Christensenellaceae R-7 group and family XIII in comparison with 80 mg/kg when analysed using the LEfSe approach [20]. This result is mainly cause by the high influence of the HTM20 group. Source (HTM vs. STM) also changed the microbial composition of the cecum. In this case, HTM groups had an increased abundance of *Streptococcus* and Enteroccocaceae family, and STM groups of family vadin BB60 group and unknown genus from Christensenellaceae family.

**Table 8:** Genera composition of the cecum higher than 1% relative abundance and genera presenting significant differences at day 34 (*n* = 12) of the birds fed with the experimental diets (hydroxychloride trace minerals, HTM and sulphate trace minerals, STM) in high (80 mg/kg) and low (20 mg/kg) dosages

	STM		HTM	
	High	Low	High	Low
*Lactobacillus*	17.6 ± 5.70	17.1 ± 8.50	17.9 ± 5.50	14.7 ± 5.00
Lachnospiraceae_other	14.0 ± 3.30	15.4 ± 4.40	15.4 ± 4.60	12.1 ± 3.40
*Bifidobacterium*	11.5 ± 6.80	9.20 ± 7.40	7.90 ± 4.10	9.90 ± 5.10
*Faecalibacterium*	10.0 ± 6.70	12.7 ± 6.60	14.1 ± 5.90	12.3 ± 4.90
*Ruminococcus* torques g	8.40 ± 5.40	6.60 ± 2.90	7.20 ± 3.50	8.50 ± 5.20
*Blautia*	6.30 ± 3.30^ab^	6.30 ± 4.30^ab^	4.70 ± 1.40^b^	7.70 ± 3.10^a^
Ruminococcaceae_other	4.50 ± 1.70	3.70 ± 1.00	4.20 ± 1.40	4.50 ± 1.40
Ruminococcaceae UCG-014	3.70 ± 2.60	4.10 ± 3.20	4.70 ± 1.40	3.80 ± 1.80
*Butyricicoccus*	2.90 ± 1.80	3.50 ± 2.20	3.50 ± 2.40	3.40 ± 1.40
*Alistipes*	2.90 ± 2.00	3.10 ± 2.20	2.70 ± 2.20	2.70 ± 1.90
*Ruminiclostridium* 5	2.50 ± 1.80^ab^	0.900 ± 0.500^b^	1.90 ± 1.50^ab^	2.20 ± 1.10^a^
*Anaerostipes*	1.80 ± 2.00^ab^	2.00 ± 1.30^ab^	1.10 ± 0.500^b^	2.20 ± 1.20^a^
*Sellimonas*	1.50 ± 1.10	1.10 ± 0.400	1.00 ± 0.500	1.50 ± 0.600
*Eubacterium hallii* g	1.40 ± 1.10	1.90 ± 1.30	1.10 ± 0.700	1.50 ± 0.900
*Dorea*	1.30 ± 1.60	1.20 ± 1.30	1.60 ± 1.70	2.10 ± 1.70
Clostridiales_other	1.00 ± 0.700	1.10 ± 0.900	1.00 ± 0.500	1.50 ± 0.800
*Eubacterium coprostanoligenes*	0.930 ± 0.570	1.20 ± 0.700	0.750 ± 0.470	0.940 ± 0.590
*Subdoligranulum*	0.900 ± 0.790	1.20 ± 1.20	1.50 ± 1.00	0.990 ± 0.620
Firmicutes_other	0.790 ± 0.680	1.00 ± 1.00	1.10 ± 1.20	0.540 ± 0.550
Christensenellaceae R-7 g	0.210 ± 0.250^b^	0.280 ± 0.250^b^	0.220 ± 0.320^b^	0.630 ± 0.470^a^
*Streptococcus*	0.160 ± 0.230^b^	0.380 ± 0.570^ab^	0.870 ± 1.280^a^	0.500 ± 0.720^ab^
*Ruminococcus* 1	0.200 ± 0.340^ab^	0.170 ± 0.300^ab^	0.110 ± 0.170^b^	0.270 ± 0.260^a^
Ruminococcaceae UCG-004	0.180 ± 0.100^ab^	0.180 ± 0.120^ab^	0.160 ± 0.090^b^	0.260 ± 0.070^a^
*CHKCI002*	0.210 ± 0.080^a^	0.130 ± 0.110^b^	0.130 ± 0.060^b^	0.160 ± 0.060^ab^
*Tyzzerella*	0.140 ± 0.160^ab^	0.090 ± 0.120^b^	0.060 ± 0.060^b^	0.180 ± 0.100^a^
vadinBB60_uncultured	0.030 ± 0.040^b^	0.190 ± 0.480^ab^	0.060 ± 0.060^a^	0.060 ± 0.100^ab^
*Anaerotruncus*	0.030 ± 0.030^ab^	0.060 ± 0.050^a^	0.020 ± 0.010^b^	0.030 ± 0.060^ab^
*Tyzzerella* 3	0.010 ± 0.010^b^	0.030 ± 0.020^a^	0.020 ± 0.010^ab^	0.020 ± 0.010^ab^
*Eubacterium nodatum* g	0.010 ± 0.010^ab^	0.020 ± 0.020^ab^	0.010 ± 0.010^b^	0.020 ± 0.010^a^
Ruminococcaceae_uncultured	0.010 ± 0.014^ab^	0.004 ± 0.006^ab^	0.001 ± 0.001^b^	0.006 ± 0.006^a^
Family XIII AD3011 g	0.009 ± 0.008^ab^	0.009 ± 0.006^ab^	0.009 ± 0.008^b^	0.022 ± 0.008^a^
*Eubacterium brachy* g	0.007 ± 0.011^b^	0.015 ± 0.023^ab^	0.010 ± 0.017^b^	0.032 ± 0.018^a^
vadinBB60_other	0.006 ± 0.015^b^	0.009 ± 0.018^b^	0.031 ± 0.020^a^	0.004 ± 0.005^b^
*Pygmaiobacter*	0.006 ± 0.014^b^	0.007 ± 0.009^ab^	0.005 ± 0.009^b^	0.074 ± 0.150^a^
Family XIII UCG-001 g	0.004 ± 0.007^b^	0.014 ± 0.011^a^	0.006 ± 0.007^b^	0.017 ± 0.009^a^
vadinBB60_uncultured Firm.	0.004 ± 0.008^ab^	0.008 ± 0.018^ab^	0.014 ± 0.012^a^	0.004 ± 0.007^b^
*Brachybacterium*	0.003 ± 0.012^ab^	0.004 ± 0.011^a^	0.001 ± 0.003^ab^	0.000 ± 0.000^b^
Peptococcaceae_other	0.001 ± 0.001^ab^	0.002 ± 0.006^ab^	0.000 ± 0.001	0.002 ± 0.003^a^

^a,b^ Different superscripts inside of each row represents significant differences among treatments (*P* < 0.05)

## Discussion

The current study tested the effect of STM compared to HTM on the growth performance, carcass characteristics and gut microbiota of broiler chickens. In this comparison two different Zn levels from STM were compared to the same levels of Zn from HTM. In all diets the same level of 15 mg/kg added Cu was used in the same source as the Zn. Due to the combined use of Zn and Cu of each source, the observed effects can not directly be linked to either Zn or Cu. Vieira [21] described a possible interaction between Zn and Cu via competition for the same carrier and due to their interaction with phytate and calcium. At the relatively low levels of Cu used in the current study no differences between the mineral sources are expected. Therefore, the focus of the discussion is on Zn, while the possible interaction effect of Cu should be kept in mind.

This study showed a beneficial effect of HTM on growth performance, specifically on body weight and average daily gain. The breast meat yield was not influenced by mineral source, however only by Zn level with higher Zn resulting in a higher breast meat yield. This is in contrast to the differences reported by Olukosi et al. [13], who described a beneficial effect of HTM over STM and a beneficial effect of 20 mg/kg over 80 mg/kg Zn. In that study, the carcass results were correlated with the growth performance results, which was not the case in the current study. Olukosi et al. [13] observed an improved gain:feed in the overall study period in the birds fed HTM compared to STM, regardless of mineral level. In the current study, feeding HTM resulted in a higher gain, but due to the increased feed intake, FCR was not significantly different. The main difference between Olukosi et al.'s study [13] and the current study could be the presence of non-starch polysaccharide (NSP) degrading enzymes in the current diet, which had a higher wheat content. High wheat diets are often challenging gut health due to the high viscosity [22, 23]. Adding NSP enzymes to the diet in the current study may have caused a lower viscosity, thus a less challenging condition in the gut. These results suggested that the trace mineral sources tested here could likely behave differently in their physiological effect in the gastro-intestinal tract (GIT). The Zn contents in ileal and cecal digesta seemed to confirm this different behaviour by the two mineral sources. The main difference was observed in the cecum between the birds fed either 80 or 20 mg/kg Zn from HTM. The higher Zn level in the cecum in the birds fed 80 mg/kg Zn from HTM suggested more available Zn later in the intestine. Although the role of the cecum in Zn uptake is not described yet, it is known that most controlled uptake of Zn takes place in the ileum [24]. Not only more Zn is present, HTM are also defined as more bioavailable for the animal compared to STM [7]. Another important characteristic of HTM is related to their crystalline structure [25]. This structure results in a low solubility above pH 4 [9], possibly leading to a more gradual release throughout the gastrointestinal tract. As a result, more minerals are available later in the intestine of HTM-fed birds. This study confirmed that more Zn was present in the lower part of the gastrointestinal tract when 80 mg/kg HTM was fed. The absorption of Zn was not measured in this study, so the uptake could not be confirmed. In the 20 mg/kg Zn groups it may be expected that the body reserves for minerals were not exceeded, resulting in less excretion of Zn back into the intestine via the gallbladder. With a higher bioavailability of HTM, less Zn would be expected in the gut contents in birds fed 20 mg/kg Zn from HTM compared to 20 mg/kg Zn from STM. The difference in Zn content of digesta may also explain the differences observed in microbiota analysis. In addition, the higher Cu content in both HTM treatment groups in the ileum indicate that by changing the source of both Zn and Cu may have synergistic effects on the birds. Future studies should focus on identifying possible interaction and synergistic effects of Cu and Zn and the influence of mineral sources on this.

Generally, the main organism present in the ileum was *Lactobacillus*. This is in line with the study of Munyaka et al. [23], who also described *Lactobacillus* to be the most abundant species in the ileum of birds fed high fiber diets. In the cecum the main species on family level were Lachnospiraceae, Ruminococcaceae, Lactobacillaceae and Bifidobacteriaceae. This is in line with Munyaka et al. [23], who observed Lachnospiraceae as the main family in the cecum. In addition to Lachnospiraceae, other families of the Clostridia such as Clostridiaceae and Ruminococcaceae are identified as main organisms in the cecum [26].

The current study shows indications for an improved richness and evenness in the diversity of cecal microbiota in birds fed HTM. In the literature, Zn is described to be important for microbiota diversity, since chronic Zn deficiency is related to a reduced species richness and diversity in the cecum [27]. Moreover, Gielda and DiRita [12] and Reed et al. [27] also revealed the importance of Zn for a proper microbiota functioning. In the present study, even though Zn levels were above current recommendations for broiler chickens the source of Zn also showed an important effect in this sense. The current results suggest that HTM is more available for both the animal, based on the performance results, and for the microbiota, based on the species diversity results.

A difference in microbiota may also be related to the growth performance of broiler chickens. Stanley et al. [28] showed a different cecal microbial community between high and low performing birds in terms of FCR. Although they suggested that cellulose and resistant starch degrading organisms were linked with high performing birds, most species could not be identified. The increase of *Blautia*, a fiber degrading organism, in ileal digesta of birds fed 80 mg/kg Zn from HTM may support this hypothesis. It is unknown whether the other different species observed in the current study may be responsible for the difference in growth performance, however the positive correlation of carcass yield and ADFI trend with Shannon and evenness indexes could support this fact.

Change in specific species of the microbiota may not always lead to a clear conclusion. For example, Clostridia are commonly found in the cecum of chickens, as shown both in laying hens and broilers [29, 30]. Similarly, *Streptococcus* species have been found in the cecum of broiler chickens [29], and depending on the species and abundance, these organisms could be beneficial for the birds. An increase in these organisms may be either beneficial or harmful. For some organisms such as *Weissella*, which was increased in the ileum of birds fed 80 mg/kg Zn from HTM, it is known that Zn has a promoting effect on it. Vahjen et al. [31] observed an increase in *Weissella* in the ileal digesta of pigs fed high levels of ZnO, showing that high Zn may increase the presence of this bacteria in the intestinal content.

Interestingly, most of the differences were observed between the groups fed 80 or 20 mg/kg Zn from HTM, whereas the STM groups reached values in between. This might suggest that HTM is able to modulate the gut microbiota in both sections of the intestine studied.

## Conclusions

Birds fed diets containing Cu and Zn from HTM performed better than birds fed minerals from STM. In the overall study, the ADG and ADFI were significantly higher in the HTM fed birds. Also a tendency towards an increased body weight was observed in HTM fed birds. This did not result in differences in carcass yield and breast meat yield. The microbial diversity in the ileum and cecum were higher in the HTM fed birds compared to the STM fed birds. Main differences in the bacterial composition were found in the cecum, especially between the group fed 80 mg/kg Zn from HTM and 20 mg/kg Zn from HTM. These were the two groups with the highest contrast in Zn levels in the cecum. Further, the main taxonomical differences found in the cecum were in between the two groups fed 80 mg/kg Zn, where the HTM group had more *Streptococcus* and two unidentified genus from vadin BB60 family and less *CHKCI002* from Eggerthellaceae family, than the STM group. Therefore, results from this study show that HTM can clearly modulate the microbiota, increase bacterial diversity in the GIT and potentially increase the growth performance in chickens.

## Data Availability

The datasets used during the current study are available from the corresponding author on reasonable request.
